# An autopsy case of vertebrobasilar dolichoectasia under hemodialysis due to autosomal dominant polycystic kidney disease

**DOI:** 10.1007/s13730-015-0190-1

**Published:** 2015-08-18

**Authors:** Shiori Nakagawa, Kengo Furuichi, Akihiro Sagara, Yasuyuki Shinozaki, Shinji Kitajima, Tadashi Toyama, Akinori Hara, Yasunori Iwata, Norihiko Sakai, Miho Shimizu, Kazuhiro Matsui, Shuichi Kaneko, Tatsuhiko Toyama, Takashi Wada

**Affiliations:** 1 Division of Nephrology, Kanazawa University Hospital, 13-1 Takara-machi, Kanazawa, 920-8641 Japan; 2 Department of Pathology, Saiseikai Takaoka Hospital, Futatsuka 387-1, Takaoka, 933-8525 Japan; 3 Division of Gastroenterology, Kanazawa University Hospital, 13-1 Takara-machi, Kanazawa, 920-8641 Japan; 4 Kouryo Clinic, Nomura 23-1, Takaoka, 933-0014 Japan; 5 Department of Laboratory Medicine, Kanazawa University Hospital, 13-1 Takara-machi, Kanazawa, 920-8641 Japan

**Keywords:** Autosomal dominant polycystic kidney disease, Vertebrobasilar dolichoectasia, Maintenance hemodialysis, Subarachnoid hemorrhage, Ischemic stroke

## Abstract

A 60-year-old male with end-stage kidney disease due to autosomal polycystic kidney disease began maintenance hemodialysis in 2005. A brain CT scan showed dilatation of left vertebral artery, basilar artery, bilateral post cerebral artery, and middle cerebral artery. At the time, he was diagnosed as vertebrobasilar dolichoectasia. He was once admitted to our hospital for ischemic stroke. After discharge, he was treated with anticoagulant agent from 2010 to 2012 without any new stroke events. In March, 2012, he was admitted to our hospital for an evaluation of diplopia and left hemiplegia. Brain MRI showed acute ischemia of bilateral pons and right temporal lobe. He was once recovered with urokinase and argatroban, but 3 months after admission, he died with sudden hypotension and impaired consciousness. Autopsy revealed that subarachnoid hemorrhage due to the rupture of basilar artery aneurysm was responsible for this event.

## Introduction

Autosomal dominant polycystic kidney disease (ADPKD), a genetic disorder with progressive cyst formation and bilateral enlarged kidneys, is sometimes associated with cardiovascular malformations, which may affect mortality of the cases. Cardiovascular manifestations of ADPKD include intracranial aneurysms, dilatation of the aortic root, dissection of the thoracic aorta and cervicocephalic arteries, coronary artery aneurysms, atrial septal aneurysms, mitral valve prolapse, and abdominal wall hernias [1, 2]. In addition to these vascular abnormalities, vertebrobasilar dolichoectasia is also one of the cardiovascular manifestations [3]. Vertebrobasilar dolichoectasia is described as marked elongation, widening, and tortuosity of arteries. Dolichoectasia is associated with hypertension, old age, and male sex. It is also reported to be associated with heritable connective tissue disorder such as Marfan syndrome and Ehlers Danlos syndrome [4]. Schievinik et al. reported the prevalence of vertebrobasilar dolichoectasia in ADPKD was 2.0–2.3 % [3]. Blood pressure control, anticoagulant agents, and antiplatelet agents are reported to prevent ischemic stroke in the vascular disease [5]. The estimated 5-year mortality is 36.2 %, and 5-year prognosis is more favorable in patients who are asymptomatic at time of diagnosis [6].

We herein report a case of dolichoectasia involving a wide range of vertebrobasilar artery with a variety of neurological symptoms.

## Case report

**Fig. 1:**
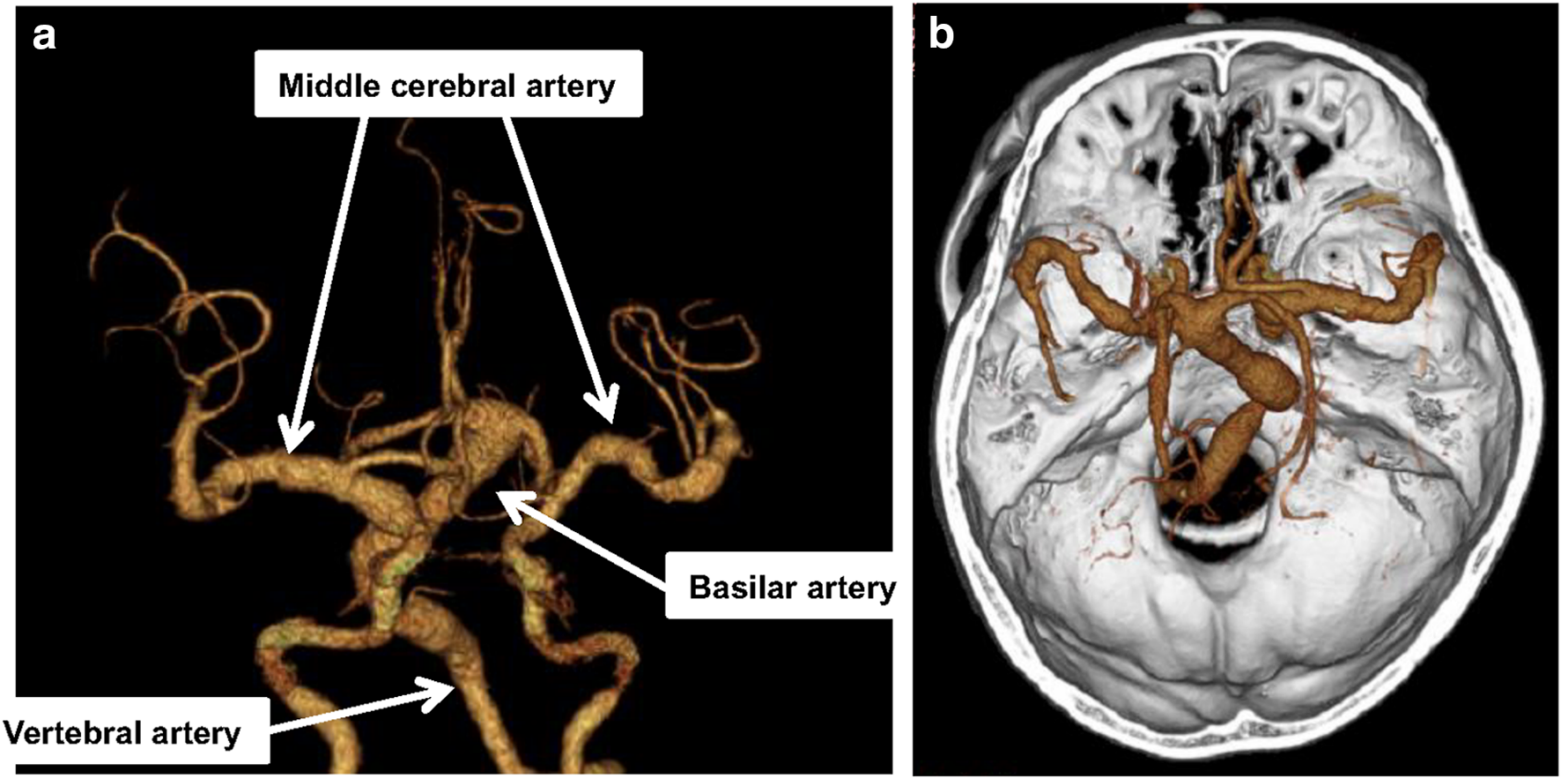
3D-CT scan image of the brain. CT scan image showed remarkable dilatation of the basilar, left vertebral, bilateral middle cerebral, and post cerebral artery. **a** Coronal view. **b** Axial view

A 60-year-old male with end-stage kidney disease due to ADPKD was admitted to our hospital for an evaluation of diplopia and left hemiplegia in March 2012. He had started maintenance hemodialysis in 2005. He had hypertension, which was relatively under good control with antihypertensive agents. He had no other arteriosclerotic risks such as diabetes, dyslipidemia, or smoking. He had no past medical history of coronary artery disease, myocardial infarction, or peripheral artery disease. The patient’s father and three brothers were also under hemodialysis due to ADPKD. None of the family members had a vascular system complication such as cerebral aneurysm, or past medical history of stroke. A brain CT scan image showed dilatation of left vertebral artery, basilar artery, bilateral post cerebral artery, and middle cerebral artery (Fig. 1a, b). At the time, he was diagnosed as vertebrobasilar dolichoectasia and started antihypertensive agents. In 2010, he was once admitted to our hospital for dysarthria, dysphagia and right hemiplegia. Brain magnetic resonance imaging (MRI) revealed acute infarction in the left midbrain, and he was started treatment with anticoagulant and additional antihypertensive agents. Although he recovered from dysarthria and dysphagia, right mild hemiplegia remained. However, he did not have any new stroke events until the admission in 2012.

On admission in 2012, his height was 163.1 cm, weight was 58.5 kg, consciousness level was 13 on the Glasgow Coma Scale, blood pressure was 146/92 mmHg, and body temperature was 36.7 °C. His conjunctiva was anemic, abdomen showed hepatomegaly and bilateral kidney swelling, and lower extremities had bilateral pitting edema. Neurological examination revealed prompt light reflection, isocoric pupil, limitation of the adduction of the right eye when gazing to the left, dysarthria, and bilateral incomplete paralysis.

**Fig. 2:**
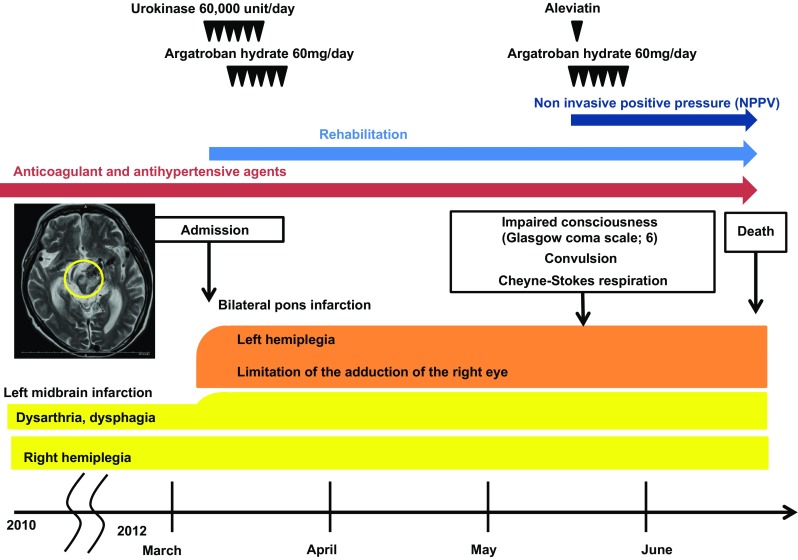
Clinical course of this case

Laboratory data were as follows: serum Cr 10.7 mg/dL, BUN 48 mg/dL, estimated glomerular filtration rate (eGFR) 4.4 mL/min/1.73 m^2^, hemoglobin (Hb) 10.7 g/dL, white blood cell count (WBC) 5700/μL, C-reactive protein (CRP) 0.4 mg/dL, aspartate aminotransferase (AST) 6 IU/L, alanine aminotransferase (ALT) 6 IU/L and alkaline phosphatase (ALP) 164 IU/L. Brain MRI showed acute ischemia of bilateral pons and right temporal lobe. Brain magnetic resonance angiography (MRA) did not show significant change in vertebrobasilar dolichoectasia compared with MRA in 2010. We started urokinase 60,000 unit/day and argatroban hydrate 60 mg/day. He was once recovered, and continued rehabilitation and maintenance hemodialysis. After 3 months of admission, he suddenly showed impaired consciousness of 6 on the Glasgow Coma Scale, convulsion, and Cheyne-Stokes respiration. CT scan image did not show any cerebral hemorrhage. Exacerbation of cerebral infarction was suspected, and we started argatroban hydrate 60 mg/day, aleviatin, and non-invasive positive pressure (NPPV). Fourteen days after convulsion, he died of sudden fall in blood pressure (Fig. 2).

**Fig. 3:**
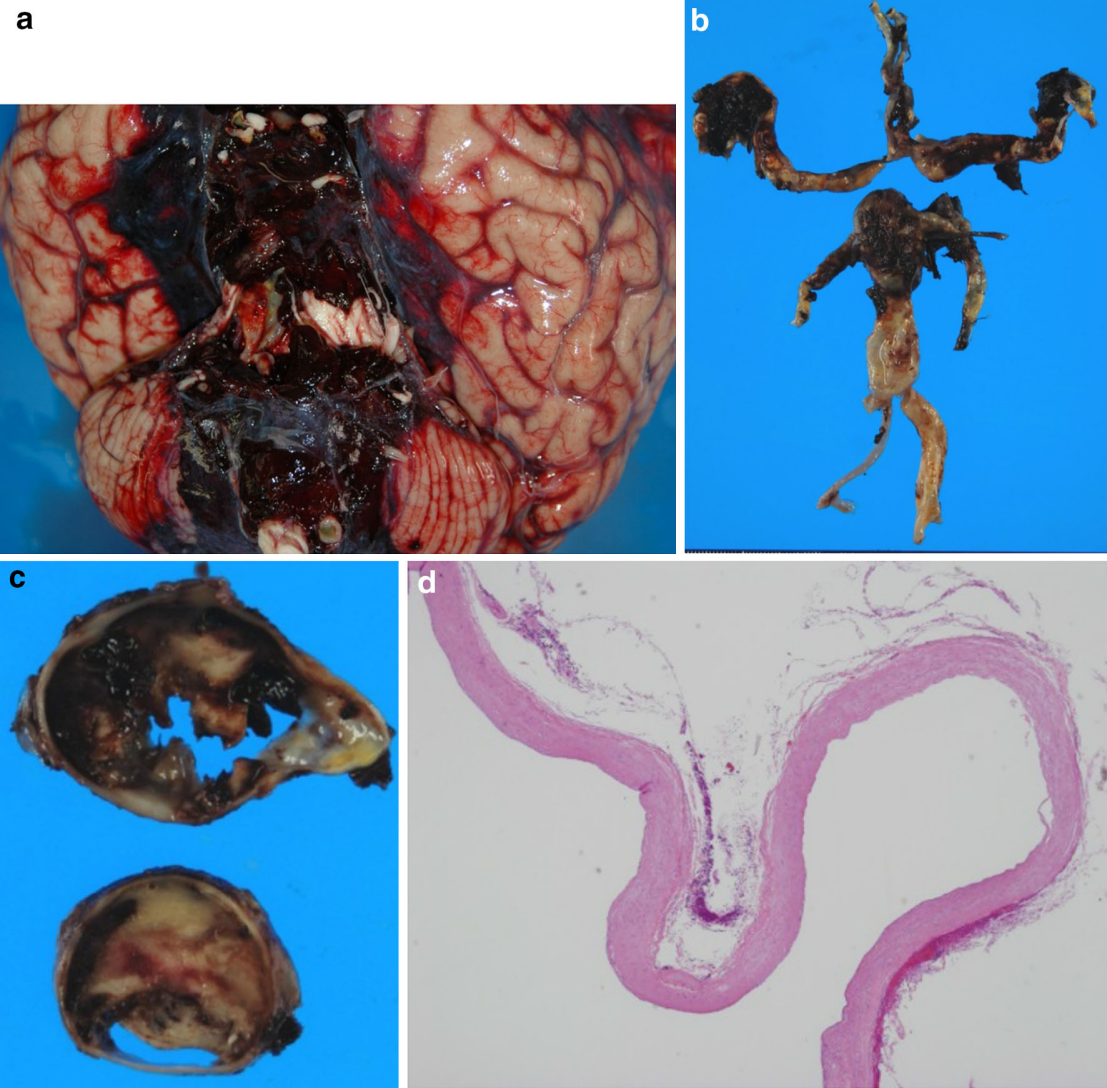
Autopsy findings of the brain. **a** Brain: massive hemorrhage in subarachnoid were seen due to rupture of basilar tip aneurysm. **b** Basilar, left vertebral and bilateral post cerebral artery showed irregular dilatation of the vessel wall. **c** Intravascular lumen of the basilar tip aneurysm was nearly obstructed by thrombi. **d** A microscopic examination of the dilatation area had rupture of the internal elastic lamina and thinning of the media

An autopsy was performed, and the brain was pathologically examined. Macroscopically, massive hemorrhage in subarachnoid were seen due to rupture of basilar tip aneurysm (Fig. 3a, b). Autopsy diagnosis of his death was subarachnoid hemorrhage. Intravascular lumen of the basilar tip aneurysm was nearly obstructed by thrombi (Fig. 3c). Old cerebral infarctions were seen in the left temporal lobe and other areas. Basilar, left vertebral, bilateral carotid, bilateral middle, and bilateral post cerebral artery showed irregular dilatation of the vessel wall. A microscopic examination of the dilatation area had rupture of the internal elastic lamina and thinning of the media (Fig. 3d). A wide range of vertebrobasilar dolichoectasia was also confirmed pathologically. In other organs, systemic arteries also showed marked atherosclerosis, and bilateral iliac artery had an aneurysm-like dilatation.

## Discussion

We report a rare vertebrobasilar dolichoectasia case with ADPKD under hemodialysis. This malformation is an uncommon vascular lesion that is found only in 0.06 % among 10,000 cerebral arteriograms, and less than 0.2 % of routine brain autopsy [7, 8]. Schievinik et al. reported the prevalence of vertebrobasilar dolichoectasia in ADPKD was 2.0–2.3 % [3]. Location of the vascular dilatation is mainly basilar artery and vertebral artery [5]. In our case, carotid artery systems were also involved, which might lead to recurring ischemic stroke and subarachnoid hemorrhage.

Histological studies show degeneration of the internal elastic lamina, multiple gaps in the internal elastic, thinning of the media secondary to reticular fiber deficiency, and smooth muscle atrophy [9]. PKD1 and PKD2 are the genes that code the proteins polycystin-1 and polycystin-2, and both are detected in vascular smooth muscle cells and endothelial cells of all major vessels. PKD2 and PKD2 interact to regulate extracellular matrix secretion or assembly, and mutations can cause altered matrix integrity which may lead to connective tissue disorder [10].

In systematic review of vertebrobasilar dolichoectasia, estimated 5-year complication risks are 17.6 % for ischemic stroke, 10.3 % for brain stem compression, 10.1 % for transient ischemic attack, 4.7 % for hemorrhagic stroke, 3.3 % for hydrocephalus, and 2.6 % for subarachnoid hemorrhage [6]. Specific treatments for vertebrobasilar dolichoectasia do not currently exist. Some studies show that blood pressure control, anticoagulant agents, and antiplatelet agents are effective for prevent ischemic stroke [5]. However, treatment with anticoagulant agents, and antiplatelet agents are also associated with an increased risk for intracerebral bleeding [11]. There are only few case reports of surgical intervention. The estimated 5-year mortality is 36.2 %, and 5-year prognosis is more favorable in patients who are asymptomatic at time of diagnosis [6]. Predictors of mortality in patients with vertebrobasilar dolichoectasia are hypertension, absence of previous warfarin use, previous anterior and posterior circulation stroke, basilar artery involvement, and progression of vertebrobasilar dolichoectasia [5, 12]. In a cohort study of vertebrobasilar dolichoectasia with lacunar stroke, vertebrobasilar dolichoectasia was not associated with risk of any of recurrent stroke, myocardial infarction, or major hemorrhage, but was associated with increased risk of death. Increased maximum basilar artery diameter was predictive of death, and maximum vertebral artery diameter showed a strong association [13]. More evidence is needed to prevent cardiovascular events of vertebrobasilar dolichoectasia.

In conclusion, we showed here a case of vertebrobasilar dolichoectasia involving a wide range of vertebrobasilar artery presenting a variety of neurological symptoms. Although vertebrobasilar dolichoectasia is an uncommon manifestation, we need to pay attention to this manifestation in ADPKD patients.
